# Diversity and distribution of ectoparasite taxa associated with *Micaelamys namaquensis* (Rodentia: Muridae), an opportunistic commensal rodent species in South Africa

**DOI:** 10.1017/S0031182022000750

**Published:** 2022-08

**Authors:** L. Stevens, A. A. Stekolnikov, E. A. Ueckermann, I. G. Horak, S. Matthee

**Affiliations:** 1 Department of Conservation Ecology and Entomology, Stellenbosch University, Private Bag X1, Matieland 7602, South Africa; 2 Zoological Institute, Russian Academy of Sciences, Saint Petersburg, Russia; 3 Unit for Environmental Sciences and Management, Potchefstroom Campus, North-West University, Private Bag X6001, Potchefstroom 2520, South Africa; 4 Department of Zoology and Entomology, Rhodes University, PO Box 94, Makhanda 6140, South Africa

**Keywords:** Distribution maps, ectoparasite diversity, *Micaelamys namaquensis*, South Africa, vectors

## Abstract

South Africa boasts a rich diversity of small mammals of which several are commensal and harbour parasites of zoonotic importance. However, limited information is available on the parasite diversity and distribution associated with rodents in South Africa. This is particularly relevant for *Micaelamys namaquensis* (Namaqua rock mouse), a regionally widespread and locally abundant species that is often commensal. To address the paucity of data, the aims of the study were to record the ectoparasite diversity associated with *M. namaquensis* and develop distribution maps of lice and mites associated with *M. namaquensis* and other rodents in South Africa. *Micaelamys namaquensis* individuals (*n* = 216) were obtained from 12 localities representing multiple biomes during 2017–2018. A total of 5591 ectoparasites representing 5 taxonomic groups – fleas, lice, mesostigmatid mites, chiggers and ticks was recorded. These consisted of at least 57 taxa of which ticks were the most speciose (20 taxa). Novel contributions include new host and locality data for several ectoparasite taxa and undescribed chigger species. Known vector species were recorded which included fleas (*Ctenocephalides felis*, *Dinopsyllus ellobius* and *Xenopsylla brasiliensis*) and ticks (*Haemaphysalis elliptica*, *Rhipicephalus appendiculatus* and *Rhipicephalus simus*). Locality records indicate within-taxon geographic differences between the 2 louse species and the 2 most abundant mite species. It is clear that *M. namaquensis* hosts a rich diversity of ectoparasite taxa and, as such, is an important rodent species to monitor in habitats where it occurs in close proximity to humans and domestic animals.

## Graphical abstract

**Figure:**
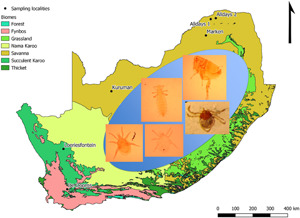

## Introduction

Small mammals, and particularly rodents, play an integral role in ecosystems, serving as both secondary consumers of seeds and other plant material (Heithaus, 1981) and as a food resource for various raptors and mesopredators (Preston, 1990; Mahmood *et al*., 2013). The Rodentia is the largest mammalian order, and species within this order have diverse biological and behavioural characteristics. Characteristics such as social structure, habitat usage and nest type are important factors that influence the exposure of rodents to ectoparasites (e.g. fleas and ticks). For example, fossorial species (e.g. mole rats) that make complex permanent underground nests generally harbour a high proportion of mites that are associated with soil and the host nest (Archer *et al*., 2014; Lutermann *et al*., 2015, 2020). Similarly, arboreal species (e.g. tree squirrels) that have limited contact with the soil surface tend to have few or no ticks which are associated with grass and low-lying vegetation (Patrick and Wilson, 1995; Romeo *et al*., 2013). Opportunism (adaptability) is another characteristic that can influence the parasite profile of a host species. Opportunistic rodent species often have large numbers of parasites due to the fact that they occupy larger geographic areas, often covering multiple vegetation types, and are able to effectively inhabit diverse land-use types (e.g. natural and transformed areas) at a local scale (Feliu *et al*., 1997; Lindenfors *et al*., 2007). The presence of rodents and their parasites in anthropogenic areas not only provides a food security risk (Muteka *et al*., 2006) but also creates opportunities for parasite transmission and disease risk in domestic animals and humans (Lecompte *et al*., 2006; Brettschneider *et al*., 2012; Mayamba *et al*., 2021). It is therefore important that parasite profiles are developed for commensal rodent species as this may identify potential disease-risk areas and facilitate sustainable disease surveillance.

South Africa is known for its rich biodiversity that includes rodents (Skinner and Chimimba, 2005). *Micaelamys namaquensis* (Namaqua rock mouse, previously *Aethomys namaquensis*) is a locally abundant and regionally widespread rodent species that is often associated with natural and anthropogenic areas (Withers *et al*., 1980; Skinner and Chimimba, 2005; Starik *et al*., 2020). The rodent occurs throughout South and southern Africa (Skinner and Chimimba, 2005; Apps, 2012; Monadjem *et al*., 2015). However, recent phylogeographic studies on *M. namaquensis* suggest biome-associated molecular differentiation in South Africa (Chimimba, 2001; Russo *et al*., 2010; Bothma *et al*., 2021). Though the species can inhabit a wide variety of biomes, it distinctly prefers rocky substrates such as outcrops, boulders and hillsides (Skinner and Chimimba, 2005; Fagir *et al*., 2014). *Micaelamys namaquensis* often uses dry grass to construct nests in rock crevices and overhangs as well as occasionally in hollows of trees (Roberts, 1951; Ansell, 1960; Choate, 1972). The species feeds on seeds, green plant material and insects (Woodall and Mackie, 1987; Kerley *et al*., 1990; Monadjem, 1997). *Micaelamys namaquensis* is described as social (Skinner and Chimimba, 2005) and makes communal nests (Choate, 1972; Flemming and Nicolson, 2004).

As yet, little is known about the ectoparasite species associated with naturally occurring rodents in South Africa. Though parasite–host and host–parasite lists are available for most rodent taxa (e.g. Zumpt, 1961; Theiler, 1962; Ledger, 1980; Segerman, 1995; Horak *et al*., 2018) the data are often outdated and in need of revision (van der Mescht and Matthee, 2017). The main critique of these reports and monographs includes incomplete sample ranges and inadequate sample sizes (Shvydka *et al*., 2018; Wilson *et al*., 2001; Stockwell and Peterson, 2002). In addition, at present there is a lack of geographic distribution maps for lice and mite species that are associated with rodents in South Africa. In recent years, empirical studies, based on larger sample sizes, have been conducted on a few rodent species (Matthee *et al*., 2007, 2010; Hillegass *et al*., 2008; Archer *et al*., 2014; Barnard *et al*., 2015; Lutermann *et al*., 2015); these include a study on *M. namaquensis* (Fagir *et al*., 2014). Although this is a step in the right direction, these studies are limited in extent as they are restricted to a single locality and/or biome. Based on current literature, *M. namaquensis* acts as a host for numerous ectoparasite taxa (Zumpt, 1961; Theiler, 1962; Ledger, 1980; Segerman, 1995; Fagir *et al*., 2014; Horak *et al*., 2018) and is also a reservoir host for vector-borne pathogens such as *Bartonella* (Brettschneider *et al*., 2012). Given the wide distribution and opportunistic behaviour of *M. namaquensis*, it is predicted that the ectoparasite diversity is currently underestimated. This prediction is supported by a recent country-wide study on flea species associated with murid rodents (van der Mescht and Matthee, 2017). To address this paucity in data, *M. namaquensis* was sampled and the ectoparasites were recorded at multiple localities and across several biomes within its distribution range in South Africa. The aim of the study was to quantify the species richness and infestation parameters of ectoparasites associated with *M. namaquensis* in South Africa. In addition, by combining the results from the current study with those of previous studies, the study aimed to provide preliminary distribution maps for the lice and more common mite species that occur on *M. namaquensis* and other rodent species in South Africa. Lastly, the development of a comprehensive ectoparasite species list provides information on the importance of *M. namaquensis* as a host for ectoparasite species of which some species are of veterinary and medical importance.

## Materials and methods

The ectoparasite material used in the study was obtained from a previous study conducted on the molecular ecology of sucking lice (Anoplura) associated with the *Aethomys*/*Micaelamys* rodent complex (Bothma *et al*., 2020, 2021).

**Fig. 1.**
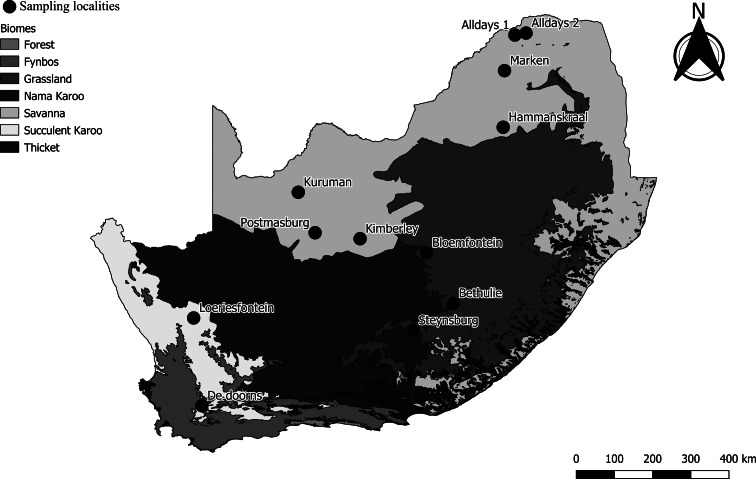
Sampling localities (*n* = 12) and biomes where *Micaelamys namaquensis* (*n* = 216) were trapped in South Africa during 2017–2018.

*Micaelamys namaquensis* individuals (*n* = 216) were trapped at 12 localities across South Africa during austral summer, autumn and winter in 2017 and 2018. The localities represented several biomes: Fynbos (1), Grassland (3), Savanna (7) and Succulent Karoo (1) (Fig. 1; Table 1). Sampling was conducted using Sherman-like live traps that were baited using a mixture of peanut butter and oats. Traps were set for 2–4 days per locality during which time they were checked twice per day. The morphological identification of *M. namaquensis* was confirmed molecularly using mitochondrial cytochrome oxidase subunit I (Bothma *et al*., 2020; S. Matthee, unpublished data).

**Table 1.** Locality information, sample size per locality and sampling period for *Micaelamys namaquensis* (*n* = 216) trapped in South Africa during 2017–2018

Location	GPS	Sample size	Sample months and year
Alldays 1	22°43′27.4″S; 28°46′36.6″E	13	December 2017
Alldays 2	22°40′00.0″S; 29°06′00.0″E	6	June 2017
Bethulie	30°30′35.3″S; 25°59′19.7″E	36	June 2017
Bloemfontein	29°02′49.1″S; 26°12′44.3″E	20	June 2018
De Doorns	33°29′00.0″S; 19°41′00.0″E	2	November 2017
Hammanskraal	25°24′00.4″S; 28°26′04.7″E	27	December 2017
Kimberley	28°38′12.0″S; 24°16′47.0″E	21	June 2018
Kuruman	27°17′15.0″S; 22°29′05.0″E	10	June 2018
Marken	23°45′27.9″S; 28°28′26.1″E	11	December 2017
Loeriesfontein	30°55′44.8″S; 19°24′02.9″E	5	April 2018
Postmasburg	28°27′46.8″S; 22°58′24.6″E	30	May 2017
Steynsburg	31°17′47.0″S; 25°49′22.0″E	35	July 2017

Only adult *M. namaquensis* (> 30 g) were included in the study. Captured individuals were placed in separate plastic bags along with a reference number and euthanized with 2–4 mL sodium pentobarbitone (200 mg kg^−1^) depending on individual body weight. After euthanasia, each individual was weighed and frozen at −20°C in the field and transferred to a −80°C freezer in the laboratory. Prior to parasite removal, the frozen carcasses were thawed and systemically examined under a stereoscopic microscope. All fleas, lice, mesostigmatid mites and ticks were removed, while only a subsample of larval trombiculid mites (chiggers) were removed with forceps. Ectoparasite taxa were placed in individual sample tubes containing 70% ethanol. Fleas, lice, mesostigmatid mites (hereafter referred to as mites) and chiggers were cleared, and the slide mounted using standard techniques, while ticks remained unmounted.

Ectoparasite identification was conducted using taxonomic reference keys. Fleas were identified according to Segerman (1995). Lice were sorted into morphospecies and a subsample from each locality was slide mounted and identified using various reference sources (e.g. Johnson, 1960; Kleynhans, 1969; Ledger, 1980; Durden and Musser, 1994). Two congeneric louse species, *Hoplopleura patersoni* and *Hoplopleura aethomydis*, share several morphological characteristics making differentiation between the 2 species troublesome. For this study the specimens were identified as *H.* cf. *patersoni*, as they share many morphological characters. However, the type specimens of these species will need to be studied to confirm whether they are indeed 2 distinct species. Due to technical difficulties not all the nymphs of the 2 lice species (*H.* cf. *patersoni* and *Polyplax praomydis*) could be identified, thus they were pooled and presented as undifferentiated nymphs. Given these restrictions, all calculations for lice species were based only on the adult stage. Mesostigmatid mites were identified according to Herrin and Tipton (1976) and Krantz and Walter (2009). The larval stage of mites in the family Trombiculidae (referred to as chiggers) was identified following Stekolnikov (2018) and the extensive taxonomic literature referenced to therein. In addition, the parasitope (body region) of the chiggers on the host was recorded at 2 localities. Ticks were identified using various reference sources (e.g. Walker *et al*., 2000; Horak *et al*., 2018). Conclusive species identifications were not possible for several ticks, notably immature stages, and they were subsequently allocated to either species groups or unknown species within a relevant genus.

The calculation of mean abundance and prevalence followed the guidelines provided in Bush *et al*. (1997). The mean parasite abundance was calculated as the total number of individuals of a particular species divided by the total number of hosts examined regardless of parasite presence. Species prevalence was calculated by recording the total number of hosts that had 1 or more individuals of a particular species present divided by the total number of hosts examined. The per-locality mean abundance and prevalence of each taxon and species were calculated similarly but the samples were restricted to these localities. Unless otherwise stated, mean abundance is presented as the mean value ± standard error. Species richness for individual parasite taxa was determined by counting the number of species present at a given locality for the parasite taxon. Only prevalence data are available for chiggers. As the chigger parasitope was not consistently recorded throughout, parasitope preference was only calculated for 2 localities (Bloemfontein and Kimberley) where the parasitope for all or the majority of samples was reported. Parasitope preferences were calculated based on chigger presence/absence on a parasitope and were reported as a percentage of chigger infestation.

## Results

A total of 5591 ectoparasites (fleas, lice, mesostigmatid mites and ticks, excluding chiggers) was recorded from 216 *M. namaquensis* individuals. At least 57 ectoparasite taxa (represent species and species groups), representing at least 26 genera, were identified (Table 2). Ticks were the most speciose taxon (20 taxa), followed by chiggers (14 species), mesostigmatid mites (11 taxa) and fleas (10 species), while lice were the most prevalent (71.76%) followed by mesostigmatid mites (50.93%) (Table 3).

**Table 2.** Ectoparasite taxa collected from *Micaelamys namaquensis* (*n* = 216) trapped across South Africa during 2017–2018

Taxon/order	Suborder	Family/subfamily	Species
Flea/Siphonaptera	Hystrichopsyllomorpha	Chimaeropsyllidae	
		Chiastopsyllinae	*Chiastopsylla godfreyi* Waterston, 1913
			*Chiastopsylla octavii* (Rothschild, 1904)
		Dinopsyllinae	*Dinopsyllus ellobius* (Rothschild, 1905)
			*Dinopsyllus lypusus* Jordan and Rothschild, 1913
		Epirimiinae	*Epirimia aganippes* (Rothschild, 1904)
		Listropsyllinae	*Listropsylla agrippinae* (Rothschild, 1904)
			*Listropsylla aricinae* de Meillon, 1949
			*Listropsylla dorippae* (Rothschild, 1904)
	Pulicomorpha	Pulicidae	
		Archaeopsyllinae	*Ctenocephalides felis* (Bouché, 1835)
		Xenopsyllinae	*Xenopsylla brasiliensis* (Baker, 1904)
Lice/Phthiraptera	Anoplura	Hoplopleuridae	*Hoplopleura* cf. *patersoni* Johnson, 1960
		Polyplacidae	*Polyplax praomydis* Bedford, 1929
Mites/Parasitiformes	Mesostigmata	Laelapidae	
		Laelapidae	*Androlaelaps dasymys* (Radford, 1939)
			*Androlaelaps rhabdomysi* Matthee and Ueckermann, 2008
			*Androlaelaps zuluensis* (Zumpt, 1950)
			*Laelaps fritzumpti* Taufflieb, 1964
			*Laelaps* aff. *grenieri*
			*Laelaps liberiensis* Hirst, 1925
			*Laelaps muricola* Träghardh, 1910
			*Laelaps vansomereni* Hirst, 1923
		Macronyssidae	Macronyssidae sp.
			*Ornithonyssus roseinnesi* (Zumpt and Till, 1953)
		Myocoptidae	*Myocoptes* sp.
Chiggers/Trombidiformes	Prostigmata	Trombiculidae	
		Gahrliepiinae	*Gahrliepia nana* (Oudemans, 1910)
		Leeuwenhoekiinae	*Hyracarus* aff. *namibiensis*
			*Hyracarus longipilosus* Lawrence, 1949
			*Hyracarus* sp.
			*Tateracarus foliosetosus* Stekolnikov and Matthee, 2022
			*Tateracarus kimberleyensis* Stekolnikov and Matthee, 2022
		Trombiculinae	*Herpetacarus gerrhosauri* (Lawrence, 1949)
			*Hypotrombidium meleagride* (Vercammen-Grandjean and Langston, 1976)
			*Kayella* sp.
			*Microtrombicula mastomyia* (Radford, 1942)
			*Microtrombicula squirreli* Stekolnikov, 2018
			*Schoutedenichia morosi* Vercammen-Grandjean, 1958
			*Schoutedenichia paraxeri* Vercammen-Grandjean, 1958
			*Schoutedenichia* sp.
Ticks/Parasitiformes	Ixodida	Ixodidae	
		Haemaphysalinae	*Haemaphysalis aciculifer-*like
			*Haemaphysalis elliptica* group
			*Haemaphysalis elliptica* (Koch, 1884)
			*Haemaphysalis elliptica*/*colesbergensis*
			*Haemaphysalis leachi* group
			*Haemaphysalis spinulosa* group
		Hyalomminae	*Hyalomma truncatum* Koch, 1844
		Ixodidae	*Ixodes bakeri*-like
		Nuttalliellidae Nuttalliellinae	*Nuttalliella* cf. *namaqua*
		Rhipicephalinae	*Rhipicephalus distinctus* Bedford, 1932
			*Rhipicephalus exophthalmos* Keirans and Walker, 1993
			*Rhipicephalus follis* Dönitz, 1910
			*Rhipicephalus follis*/*gertrudae*
			*Rhipicephalus gertrudae* Feldman-Muhsam, 1960
			*Rhipicephalus lunulatus* Neumann, 1907
			*Rhipicephalus neumanni* Walker, 1990
			*Rhipicephalus pravus* group
			*Rhipicephalus simus*/*distinctus*
			*Rhipicephalus simus*/*follis*
			*Rhipicephalus warburtoni* Walker and Horak, 2000

**Table 3.** Infestation parameters for the ectoparasite taxa recorded on *Micaelamys namaquensis* (*n* = 216) in South Africa during 2017–2018

	Ectoparasite taxa	Larvae (%)	Nymphs (%)	Adults (%)	Sex ratio (♂:♀)	Mean abundance (±s.e.)	Prevalence (%)
Fleas		**0**	**0**	**100**	**1:0.89**	**1.95 ± 0.28**	**47**.**69**
	*Chiastopsylla godfreyi*	0	0	100	1:0.72	0.53 ± 0.17	10.65
	*Chiastopsylla octavii*	0	0	100	1:1.86	0.09 ± 0.08	1.39
	*Ctenocephalides felis*	0	0	100	0:4	0.02 ± 0.01	1.85
	*Dinopsyllus ellobius*	0	0	100	1:0.64	0.08 ± 0.04	3.70
	*Dinopsyllus lypusus*	0	0	100	1:1	0.009 ± 0.01	0.93
	*Epirimia aganippes*	0	0	100	1:1.39	0.20 ± 0.06	6.94
	*Listropsylla agrippinae*	0	0	100	1:0.67	0.05 ± 0.02	2.78
	*Listropsylla aricinae*	0	0	100	1:1	0.03 ± 0.02	0.93
	*Listropsylla dorippae*	0	0	100	1:0	0.01 ± 0.01	0.46
	*Xenopsylla brasiliensis*	0	0	100	1:0.87	0.89 ± 0.13	28.24
Lice		**–**	**30.94**	**69.06**	**1:0.89**	**18.87 ± 3**	**71**.**76**
	*Hoplopleura* cf. *patersoni*^a^	**–**	**–**	100	1:1.40	2.94 ± 0.46	43.06
	*Polyplax praomydis*^a^	**–**	**–**	100	1:0.75	7.48 ± 1.11	47.69
Mites		**0.34**	**31.45**	**68.21**	**1:3.59**	**2.70 ± 0.4**	**50**.**93**
	*Androlaelaps dasymys*	0	25	75	1:7	0.05 ± 0.04	0.93
	*Androlaelaps rhabdomysi*	0.38	42.05	57.58	1:2.30	1.22 ± 0.28	25.46
	*Androlaelaps zuluensis*	0	0	100	1:2	0.01 ± 0.01	1.39
	*Laelaps fritzumpti*	0	10.40	89.60	1:6.05	0.80 ± 0.14	24.07
	*Laelaps* aff. *grenieri*	1.03	43.30	55.67	1:3.91	0.45 ± 0.22	10.65
	*Laelaps liberiensis*	0	0	100	1:3.5	0.04 ± 0.02	1.39
	*Laelaps muricola*	0	23.08	76.92	1:2.33	0.06 ± 0.04	1.39
	*Laelaps vansomereni*	0	0	100	0:5	0.02 ± 0.02	0.93
	Macronyssidae sp.	0	75	25	1:0	0.02 ± 0.01	0.93
	*Ornithonyssus roseinnesi*	0	83.33	16.67	0:1	0.03 ± 0.02	1.39
	*Myocoptes* sp.	0	0	100	0:1	0.01 ± 0.01	0.46
Chiggers		**100**	**–**	**–**	**–**	**–**	**41**.**20**
	*Gahrliepia nana*	100	**–**	**–**	**–**	**–**	0.93
	*Herpetacarus gerrhosauri*	100	**–**	**–**	**–**	**–**	1.39
	*Hypotrombidium meleagride*	100	**–**	**–**	**–**	**–**	1.39
	*Hyracarus* aff. *namibiensis*	100	**–**	**–**	**–**	**–**	20.37
	*Hyracarus longipilosus*	100	**–**	**–**	**–**	**–**	7.87
	*Hyracarus* sp.	100	**–**	**–**	**–**	**–**	0.46
	*Kayella* sp.	100	**–**	**–**	**–**	**–**	5.09
	*Microtrombicula mastomyia*	100	**–**	**–**	**–**	**–**	0.93
	*Microtrombicula squirreli*	100	**–**	**–**	**–**	**–**	0.46
	*Schoutedenichia morosi*	100	**–**	**–**	**–**	**–**	3.70
	*Schoutedenichia paraxeri*	100	**–**	**–**	**–**	**–**	0.46
	*Schoutedenichia* sp.	100	**–**	**–**	**–**	**–**	0.93
	*Tateracarus foliosetosus*	100	**–**	**–**	**–**	**–**	2.31
	*Tateracarus kimberleyensis*	100	**–**	**–**	**–**	**–**	0.93
Ticks		**74.95**	**24.66**	**0.39**	**0:2**	**2.38 ± 0.38**	**50**.**00**
	*Haemaphysalis aciculifer-*like	100	0	0	0	0.15 ± 0.09	2.78
	*Haemaphysalis elliptica* group	92.86	7.14	0	0	0.06 ± 0.03	2.78
	*Haemaphysalis elliptica*	6.25	93.75	0	0	0.07 ± 0.02	5.56
	*Haemaphysalis elliptica/colesbergensis*	0	100	0	0	0.03 ± 0.03	0.46
	*Haemaphysalis leachi* group	75.00	25	0	0	0.07 ± 0.04	1.85
	*Haemaphysalis spinulosa* group	76.8	23.2	0	0	0.58 ± 0.20	15.74
	*Haylomma truncatum*	80.00	20	0	0	0.05 ± 0.02	4.63
	*Ixodes bakeri*-like	73.17	21.95	4.89	0:2	0.19 ± 0.05	7.87
	*Nuttalliella* cf. *namaqua*	100	0	0	0	0.01 ± 0.01	0.46
	*Rhipicephalus exophthalmos*	94.44	5.56	0	0	0.08 ± 0.03	4.63
	*Rhipicephalus distinctus*	100	0	0	0	0.01 ± 0.01	0.46
	*Rhipicephalus follis*	14.29	85.71	0	0	0.03 ± 0.01	2.78
	*Rhipicephalus follis/gertrudae*	100	0	0	0	0.01 ± 0.01	0.46
	*Rhipicephalus gertrudae*	66.67	33.33	0	0	0.01 ± 0.01	0.93
	*Rhipicephalus lunulatus*	0	100	0	0	0.03 ± 0.02	1.85
	*Rhipicephalus neumanni*	96.30	3.7	0	0	0.24 ± 0.14	2.78
	*Rhipicephalus pravus* group	100	0	0	0	0.01 ± 0.01	0.46
	*Rhipicephalus simus*/*follis*	72.37	27.63	0	0	0.70 ± 0.23	11.11
	*Rhipicephalus simus*/*distinctus*	75.00	25	0	0	0.02 ± 0.01	1.39
	*Rhipicephalus warburtoni*	100	0	0	0	0.01 ± 0.01	1.39

Bold text indicate the values at the higher taxonomic level.

a Data based on adult life stage only.

Ten flea species, representing 6 genera, were recorded on *M. namaquensis* (Table 2). Overall, *Xenopsylla brasiliensis* was the most abundant and prevalent species (0.89 ± 0.13, 28.24%, respectively) followed by *Chiastopsylla godfreyi* (0.53 ± 0.17, 10.65%) (Table 3). *Xenopsylla brasiliensis* was recorded at 7 localities (Table 4) and was present on ≥10% of the rodents at each locality (Table 5). *Chiastopsylla godfreyi* and *Dinopsyllus ellobius* were more restricted in their distribution (4 localities) and less prevalent compared to *X. brasiliensis* (Tables 4 and 5). Female-biased flea infestations were only recorded for 3 species (*Chiastopsylla octavii*, *Ctenocephalides felis* and *Epirimia aganippes*) (Table 3), while male-biased sex ratios were recorded for *C. godfreyi* and *X. brasiliensis* at several localities (Supplementary Table 1).

**Table 4.** Mean abundance (±s.e.) and occurrence per locality of individual ectoparasite taxa recovered from *Micaelamys namaquensis* (*n* = 216) across South Africa during 2017–2018

Parasite	Species	Occurrence	Alldays 1	Alldays 2	Bethulie	Bloem-fontein	De Doorns	Hammans-kraal	Kim-berley	Kuruman	Loeries-fontein	Marken	Postmas-burg	Steyns-burg
Fleas														
	*Chiastopsylla godfreyi*	4	**–**	**–**	1.11 ± 0.57	1.45 ± 0.80	**–**	**–**	0.29 ± 0.24	**–**	**–**	**–**	**–**	1.14 ± 0.72
	*Chiastopsylla octavii*	2	**–**	**–**	**–**	0.85 ± 0.85	**–**	**–**	**–**	**–**	**–**	**–**	**–**	0.09 ± 0.06
	*Ctenocephalides felis*	2	**–**	**–**	0.08 ± 0.05	**–**	**–**	**–**	**–**	**–**	**–**	**–**	0.03 ± 0.03	**–**
	*Dinopsyllus ellobius*	4	0.33 ± 0.21	**–**	**–**	0.35 ± 0.35	**–**	0.19 ± 0.15	0.19 ± 0.11	**–**	**–**	**–**	**–**	**–**
	*Dinopsyllus lypusus*	2	**–**	**–**	**–**	**–**	**–**	0.04 ± 0.04	0.05 ± 0.05	**–**	**–**	**–**	**–**	**–**
	*Epirimia aganippes*	3	4.5 ± 0.99	**–**	0.25 ± 0.12	**–**	**–**	**–**	**–**	**–**	**–**	**–**	**–**	0.2 ± 0.12
	*Listropsylla agrippinae*	3	**–**	**–**	0.06 ± 0.04	0.2 ± 0.16	**–**	**–**	**–**	**–**	**–**	**–**	**–**	0.11 ± 0.08
	*Listropsylla aricinae*	1	**–**	**–**	**–**	**–**	**–**	**–**	0.29 ± 0.24	**–**	**–**	**–**	**–**	**–**
	*Listropsylla dorippae*	1	**–**	**–**	**–**	**–**	**–**	**–**	0.05 ± 0.05	**–**	**–**	**–**	**–**	**–**
	*Xenopsylla brasiliensis*	7	0.33 ± 0.33	2.38 ± 0.72	**–**	0.15 ± 0.11	**–**	1.96 ± 0.40	3 ± 0.75	**–**	**–**	0.73 ± 0.36	1.2 ± 0.41	**–**
Lice														
	*Hoplopleura* cf. *patersoni*^a^	10	1.83 ± 1.11	6.46 ± 2.97	2.89 ± 1.16	5.45 ± 2.37	**–**	1.81 ± 0.81	0.05 ± 0.05	0.4 ± 0.4	**–**	5.55 ± 3.18	2.3 ± 0.98	4.09 ± 1.12
	*Polyplax praomydis*^a^	9	**–**	1 ± 0.52	15.28 ± 4.07	0.55 ± 0.55	3 ± 2	**–**	3.67 ± 1.09	1.8 ± 0.9	3 ± 2.76	**–**	4.93 ± 1.09	22.2 ± 4.02
Mites														
	*Androlaelaps dasymys*	1	**–**	**–**	**–**	**–**	**–**	0.37 ± 0.30	**–**	**–**	**–**	**–**	**–**	**–**
	*Androlaelaps rhabdomysi*	9	**–**	0.08 ± 0.05	2.78 ± 1.30	0.35 ± 0.24	1 ± 1	**–**	1 ± 0.38	2.2 ± 0.84	**–**	0.18 ± 0.12	0.63 ± 0.27	2.57 ± 0.90
	*Androlaelaps zuluensis*	2	**–**	**–**	**–**	**–**	**–**	**–**	**–**	**–**	**–**	**–**	0.07 ± 0.05	0.03 ± 0.03
	*Laelaps fritzumpti*	8	**–**	1.62 ± 0.84	1.56 ± 0.43	0.15 ± 0.11	**–**	0.04 ± 0.04	2.43 ± 0.70	**–**	**–**	0.27 ± 0.19	0.4 ± 0.19	0.74 ± 0.39
	*Laelaps* aff. *grenieri*	5	**–**	**–**	1.31 ± 1.17	0.5 ± 0.22	**–**	1.22 ± 0.85	0.14 ± 0.10	**–**	**–**	**–**	**–**	0.11 ± 0.15
	*Laelaps liberiensis*	2	**–**	**–**	**–**	0.15 ± 0.15	**–**	0.22 ± 0.15	**–**	**–**	**–**	**–**	**–**	**–**
	*Laelaps muricola*	1	**–**	**–**	**–**	**–**	**–**	0.48 ± 0.31	**–**	**–**	**–**	**–**	**–**	**–**
	*Laelaps vansomereni*	2	**–**	**–**	**–**	**–**	1 ± 1	**–**	**–**	**–**	**–**	0.27 ± 0.27	**–**	**–**
	Macronyssidae sp.	1	**–**	0.31 ± 0.21	**–**	**–**	**–**	**–**	**–**	**–**	**–**	**–**	**–**	**–**
	*Ornithonyssus roseinnesi*	3	**–**	0.23 ± 0.23	**–**	**–**	**–**	**–**	**–**	**–**	0.4 ± 0.40	0.09 ± 0.09	**–**	**–**
	*Myocoptes* sp.	1	**–**	**–**	**–**	**–**	**–**	0.04 ± 0.04	**–**	**–**	**–**	**–**	**–**	**–**
Ticks														
	*Haemaphysalis aciculifer-*like	1	**–**	**–**	**–**	**–**	**–**	1.19 ± 0.66	**–**	**–**	**–**	**–**	**–**	**–**
	*Haemaphysalis elliptica* group	1	**–**	**–**	**–**	0.7 ± 0.24	**–**	**–**	**–**	**–**	**–**	**–**	**–**	**–**
	*Haemaphysalis elliptica*	3	**–**	**–**	**–**	0.15 ± 0.11	**–**	0.15 ± 0.12	**–**	**–**	**–**	**–**	**–**	0.26 ± 0.09
	*Haemaphysalis elliptica/colesbergensis*	1	**–**	**–**	**–**	**–**	3 ± 3	**–**	**–**	**–**	**–**	**–**	**–**	**–**
	*Haemaphysalis leachi* group	2	1.33 ± 0.99	**–**	**–**	0.4 ± 0.31	**–**	**–**	**–**	**–**	**–**	**–**	**–**	**–**
	*Haemaphysalis spinulosa* group	7	0.33 ± 0.33	**–**	2.03 ± 1.05	0.35 ± 0.35	**–**	1 ± 0.30	**–**	1.4 ± 1.19	0.2 ± 0.20	0.18 ± 0.18	**–**	**–**
	*Haylomma truncatum*	3	**–**	**–**	**–**	**–**	**–**	**–**	0.38 ± 0.13	0.2 ± 0.13	0.2 ± 0.20	**–**	**–**	**–**
	*Ixodes bakeri-*like	3	0.17 ± 0.33	**–**	**–**	1.85 ± 0.41	**–**	**–**	**–**	**–**	**–**	0.18 ± 0.18	**–**	**–**
	*Nuttalliella* cf. *namaqua*	1	**–**	**–**	**–**	**–**	**–**	**–**	**–**	**–**	0.2 ± 0.20	**–**	**–**	**–**
	*Rhipicephalus exophthalmos*	2	**–**	**–**	**–**	**–**	**–**	**–**	**–**	**–**	**–**	**–**	0.57 ± 0.19	0.03 ± 0.03
	*Rhipicephalus distinctus*	1	**–**	**–**	0.08 ± 0.08	**–**	**–**	**–**	**–**	**–**	**–**	**–**	**–**	**–**
	*Rhipicephalus follis*	4	**–**	**–**	0.06 ± 0.04	0.1 ± 0.10	**–**	0.04 ± 0.04	**–**	**–**	**–**	**–**	**–**	0.06 ± 0.04
	*Rhipicephalus follis*/*gertrudae*	1	**–**	**–**	**–**	**–**	**–**	0.04 ± 0.04	**–**	**–**	**–**	**–**	**–**	**–**
	*Rhipicephalus gertrudae*	1	**–**	**–**	**–**	0.15 ± 0.11	**–**	**–**	**–**	**–**	**–**	**–**	**–**	**–**
	*Rhipicephalus lunulatus*	1	**–**	**–**	**–**	**–**	**–**	**–**	**–**	**–**	**–**	0.64 ± 0.36	**–**	**–**
	*Rhipicephalus neumanni*	1	**–**	**–**	**–**	**–**	**–**	**–**	**–**	5.2 ± 2.68	**–**	**–**	**–**	**–**
	*Rhipicephalus pravus* group	1	**–**	**–**	0.03 ± 0.03	**–**	**–**	**–**	**–**	**–**	**–**	**–**	**–**	**–**
	*Rhipicephalus simus*/*distinctus*	1	**–**	**–**	**–**	**–**	**–**	**–**	**–**	0.4 ± 0.22	**–**	**–**	**–**	**–**
	*Rhipicephalus simus*/*follis*	4	0.17 ± 0.17	**–**	**–**	**–**	**–**	2.30 ± 1.15	**–**	**–**	**–**	7.91 ± 2.77	**–**	0.06 ± 0.04
	*Rhipicephalus warburtoni*	3	0.17 ± 0.17	**–**	**–**	**–**	**–**	0.04 ± 0.04	**–**	**–**	**–**	**–**	**–**	0.03 ± 0.03

a Data based on adult life stage only.

**Table 5.** Per locality prevalence (%) of individual ectoparasite taxa recovered from *Micaelamys namaquensis* (*n* = 216) across South Africa during 2017–2018

Parasite	Species	Alldays 1	Alldays 2	Bethulie	Bloemfontein	De Doorns	Hammanskraal	Kimberley	Kuruman	Loeriesfontein	Marken	Postmasburg	Steynsburg
Fleas													
	*Chiastopsylla godfreyi*	**–**	**–**	25	25	**–**	**–**	9.52	**–**	**–**	**–**	**–**	20
	*Chiastopsylla octavii*	**–**	**–**	**–**	5	**–**	**–**	**–**	**–**	**–**	**–**	**–**	5.71
	*Ctenocephalides felis*	**–**	**–**	8.33	**–**	**–**	**–**	**–**	**–**	**–**	**–**	3.33	**–**
	*Dinopsyllus ellobius*	33.33	**–**	**–**	5	**–**	7.41	14.29	**–**	**–**	**–**	**–**	**–**
	*Dinopsyllus lypusus*	**–**	**–**	**–**	**–**	**–**	3.7	4.76	**–**	**–**	**–**	**–**	**–**
	*Epirimia aganippes*	100	**–**	13.89	**–**	**–**	**–**	**–**	**–**	**–**	**–**	**–**	11.43
	*Listropsylla agrippinae*	**–**	**–**	5.56	10	**–**	**–**	**–**	**–**	**–**	**–**	**–**	5.71
	*Listropsylla aricinae*	**–**	**–**	**–**	**–**	**–**	**–**	9.52	**–**	**–**	**–**	**–**	**–**
	*Listropsylla dorippae*	**–**	**–**	**–**	**–**	**–**	**–**	4.76	**–**	**–**	**–**	**–**	**–**
	*Xenopsylla brasiliensis*	16.67	69.23	**–**	10	**–**	62.96	76.19	**–**	**–**	36.36	40	**–**
Lice													
	*Hoplopleura* cf. *patersoni*^a^	50	61.54	50	80	**–**	29.63	4.76	10	**–**	54.55	40	57.14
	*Polyplax praomydis*^a^	**–**	30.77	75	5	100	**–**	71.43	50	40	**–**	60	82.86
Mites													
	*Androlaelaps dasymys*	**–**	**–**	**–**	**–**	**–**	7.41	**–**	**–**	**–**	**–**	**–**	**–**
	*Androlaelaps rhabdomysi*	**–**	7.69	36.11	10	50	**–**	38.1	50	**–**	18.18	26.67	42.86
	*Androlaelaps zuluensis*	**–**	**–**	**–**	**–**	**–**	**–**	**–**	**–**	**–**	**–**	6.67	2.86
	*Laelaps fritzumpti*	**–**	30.77	47.22	10	**–**	3.7	61.9	**–**	**–**	18.18	20	20
	*Laelaps* aff. *grenieri*	**–**	**–**	11.11	30	**–**	33.33	9.52	**–**	**–**	**–**	**–**	5.71
	*Laelaps liberiensis*	**–**	**–**	**–**	5	**–**	7.41	**–**	**–**	**–**	**–**	**–**	**–**
	*Laelaps muricola*	**–**	**–**	**–**	**–**	**–**	11.11	**–**	**–**	**–**	**–**	**–**	**–**
	*Laelaps vansomereni*	**–**	**–**	**–**	**–**	50	**–**	**–**	**–**	**–**	9.09	**–**	**–**
	Macronyssidae sp.	**–**	15.38	**–**	**–**	**–**	**–**	**–**	**–**	**–**	**–**	**–**	**–**
	*Ornithonyssus roseinnesi*	**–**	7.69	**–**	**–**	**–**	**–**	**–**	**–**	20	9.09	**–**	**–**
	*Myocoptes* sp.	**–**	**–**	**–**	**–**	**–**	3.7	**–**	**–**	**–**	**–**	**–**	**–**
Chiggers													
	*Gahrliepia nana*	**–**	**–**	**–**	**–**	**–**	**–**	9.52	**–**	**–**	**–**	**–**	**–**
	*Herpetacarus gerrhosauri*	**–**	**–**	**–**	**–**	**–**	**–**	**–**	**–**	**–**	**–**	**–**	8.57
	*Hypotrombidium meleagride*	**–**	**–**	**–**	**–**	**–**	**–**	**–**	**–**	60	**–**	**–**	**–**
	*Hyracarus* aff. *namibiensis*	**–**	**–**	77.78	45	**–**	**–**	**–**	10	**–**	**–**	**–**	20
	*Hyracarus longipilosus*	**–**	**–**	**–**	**–**	**–**	**–**	**–**	**–**	**–**	**–**	**–**	48.57
	*Hyracarus* sp.	**–**	**–**	**–**	**–**	50	**–**	**–**	**–**	**–**	**–**	**–**	**–**
	*Kayella* sp.	**–**	**–**	**–**	**–**	**–**	**–**	**–**	**–**	**–**	**–**	**–**	31.43
	*Microtrombicula mastomyia*	**–**	**–**	**–**	**–**	**–**	7.41	**–**	**–**	**–**	**–**	**–**	**–**
	*Microtrombicula squirreli*	**–**	7.69	**–**	**–**	**–**	**–**	**–**	**–**	**–**	**–**	**–**	**–**
	*Schoutedenichia morose*	**–**	7.69	**–**	25	**–**	**–**	**–**	**–**	**–**	**–**	**–**	5.71
	*Schoutedenichia paraxeri*	**–**	7.69	**–**	**–**	**–**	**–**	**–**	**–**	**–**	**–**	**–**	**–**
	*Schoutedenichia* sp.	**–**	**–**	**–**	**–**	**–**	**–**	**–**	**–**	40	**–**	**–**	**–**
	*Tateracarus foliosetosus*	**–**	**–**	**–**	**–**	**–**	**–**	**–**	50	**–**	**–**	**–**	**–**
	*Tateracarus kimberleyensis*	**–**	**–**	**–**	**–**	**–**	**–**	52.38	**–**	**–**	**–**	**–**	**–**
Ticks													
	*Haemaphysalis aciculifer-*like	**–**	**–**	**–**	**–**	**–**	22.22	**–**	**–**	**–**	**–**	**–**	**–**
	*Haemaphysalis elliptica*	**–**	**–**	**–**	10	**–**	7.41	**–**	**–**	**–**	**–**	**–**	22.86
	*Haemaphysalis elliptica* group	**–**	**–**	**–**	30	**–**	**–**	**–**	**–**	**–**	**–**	**–**	**–**
	*Haemaphysalis elliptica/colesbergensis*	**–**	**–**	**–**	**–**	50	**–**	**–**	**–**	**–**	**–**	**–**	**–**
	*Haemaphysalis leachi* group	33.33	**–**	**–**	10	**–**	**–**	**–**	**–**	**–**	**–**	**–**	**–**
	*Haemaphysalis spinulosa* group	16.67	**–**	50	5	**–**	37.04	**–**	20	20	9.09	**–**	**–**
	*Hyalomma truncatum*	**–**	**–**	**–**	**–**	**–**	**–**	33.33	20	20	**–**	**–**	**–**
	*Ixodes bakeri-*like	16.67	**–**	**–**	75	**–**	**–**	**–**	**–**	**–**	9.09	**–**	**–**
	*Nuttalliella* cf. *namaqua*	**–**	**–**	**–**	**–**	**–**	**–**	**–**	**–**	20	**–**	**–**	**–**
	*Rhipicephalus distinctus*	**–**	**–**	2.78	**–**	**–**	**–**	**–**	**–**	**–**	**–**	**–**	**–**
	*Rhipicephalus exophthalmos*	**–**	**–**	**–**	**–**	**–**	**–**	**–**	**–**	**–**	**–**	30	2.86
	*Rhipicephalus follis*	**–**	**–**	5.56	5	**–**	3.7	**–**	**–**	**–**	**–**	**–**	5.71
	*Rhipicephalus follis*/*gertrudae*	**–**	**–**	**–**	**–**	**–**	3.7	**–**	**–**	**–**	**–**	**–**	**–**
	*Rhipicephalus gertrudae*	**–**	**–**	**–**	10	**–**	**–**	**–**	**–**	**–**	**–**	**–**	**–**
	*Rhipicephalus lunulatus*	**–**	**–**	**–**	**–**	**–**	**–**	**–**	**–**	**–**	36.36	**–**	**–**
	*Rhipicephalus neumanni*	**–**	**–**	**–**	**–**	**–**	**–**	**–**	60	**–**	**–**	**–**	**–**
	*Rhipicephalus pravus* group	**–**	**–**	2.78	**–**	**–**	**–**	**–**	**–**	**–**	**–**	**–**	**–**
	*Rhipicephalus simus*/*distinctus*	**–**	**–**	**–**	**–**	**–**	**–**	**–**	30	**–**	**–**	**–**	**–**
	*Rhipicephalus simus*/*follis*	16.67	**–**	**–**	**–**	**–**	51.85	**–**	**–**	**–**	63.64	**–**	5.71
	*Rhipicephalus warburtoni*	16.67	**–**	**–**	**–**	**–**	3.7	**–**	**–**	**–**	**–**	**–**	2.86

a Data based on adult life stage only.

**Fig. 2.**
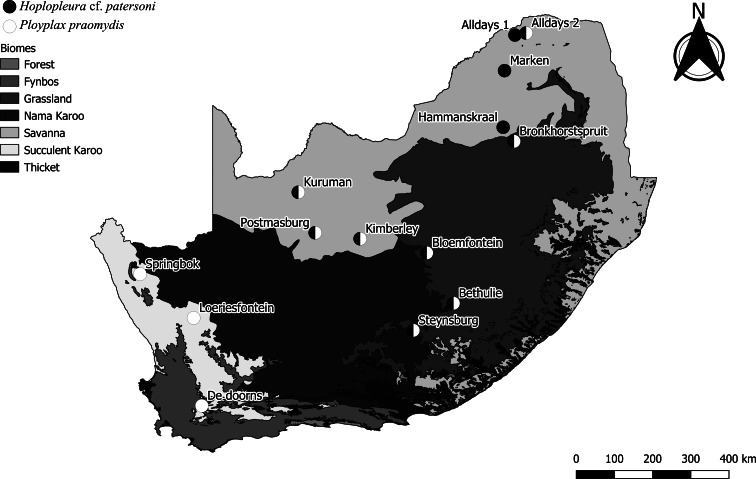
Occurrence records (*n* = 14) for *Hoplopleura* cf. *patersoni* and *Polyplax praomydis* recorded from *Micaelamys namaquensis* in South Africa (data from the present study; Fagir *et al*., 2014; Bothma *et al*., 2020, 2021).

Two louse species, *H.* cf. *patersoni* and *P. praomydis*, were recorded (Table 2). The following data are based on adult stages only. The overall mean abundance and prevalence of *H.* cf. *patersoni* were lower (2.94 ± 0.46, 43.06%) than *P. praomydis* (7.48 ± 1.11, 47.69%) (Table 3). *Hoplopleura* cf. *patersoni* was present at 10 localities and *P. praomydis* at 9, while the 2 species co-occurred at 7 of the 12 localities (Table 4). Apart from an outlier (Alldays 2), *P. praomydis* was collected at the central and western localities, while *H.* cf. *patersoni* was recovered at the central and north-eastern localities (Fig. 2). *Polyplax praomydis* was more abundant and prevalent than *H.* cf. *patersoni* at 5 of the 6 central localities where they co-occurred (Tables 4 and 5). In both species the sex ratio was variable between localities (Supplementary Table 2).

**Fig. 3.**
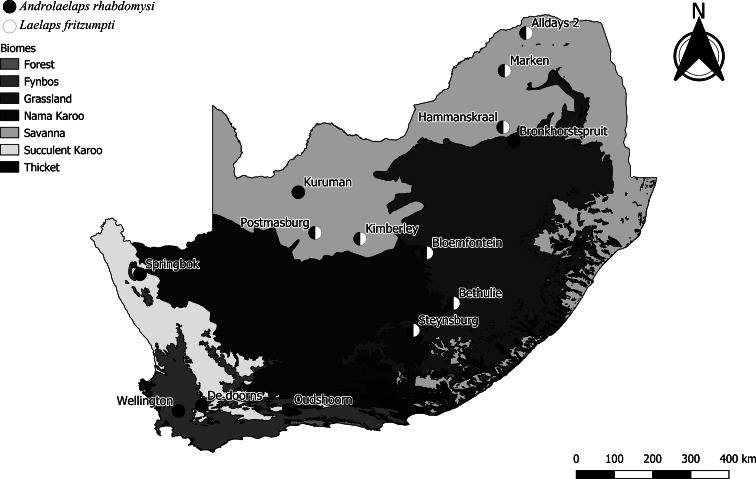
Occurrence record (*n* = 14) for *Androlaelaps rhabdomysi* and *Laealaps fritzumpti* recorded from *Micaelamys namaquensis* and *Rhabdomys pumilio* (data from the present study; Matthee and Ueckermann, 2008; Fagir *et al*., 2014; S. Matthee, unpublished data).

A total of 11 mesostigmatid mite species was collected from *M. namaquensis* (Table 2). *Androlaelaps rhabdomysi* was the most abundant and prevalent species (1.22 ± 0.28, 25.46%) followed by *Laelaps fritzumpti* (0.80 ± 0.14, 24.07%) (Table 3). *Androlaelaps rhabdomysi* was recorded at 9 localities and *L. fritzumpti* at 8 (Table 4). *Androlaelaps rhabdomysi* was present across South Africa, while *L. fritzumpti* occurred in the central and north-eastern localities (Fig. 3). Female-biased infestations were recorded for 10 mite species (Table 3) and this pattern was common across localities (Supplementary Table 3).

A total of 14 chigger species was recorded from *M. namaquensis* (Table 2). The chiggers represent 10 known species, 2 new undescribed species (*Kayella* sp. and *Schoutedenichia* sp.) and 2 potentially new species that require additional examination (*Hyracarus* sp. and *Hyracarus* aff. *namibiensis*). New localities were recorded for 5 chigger species. Only 2 species occurred at multiple localities: *H.* aff. *namibiensis* was present at 4 localities, followed by *Schoutedenichia morosi* at 3 localities (Table 5). The per-locality species richness varied among localities with 5 species recorded at Steynsburg followed by 3 at Alldays 2 (Table 5). The per-locality prevalence of chiggers varied between 7.69% (Alldays 2) and 77.78% (Bethulie). Chiggers were recorded from 6 parasitopes on the host (body, ear, genital area, head, leg and tail base) of which the ear was one of the preferred parasitopes at both Bloemfontein (71.43%) and Kimberley (72.73%) (Table 6). In addition, the tail base was also one of the preferred parasitopes (71.43 and 27.27%, respectively) (Table 6).

**Table 6.** Summary of parasitope preference, presented as prevalence (%), for chiggers recovered from *Micaelamys namaquensis* (*n* = 41) at 2 localities in South Africa during June 2018

	Parasitope					
Locality	Body	Ear	Genital area	Head	Leg	Tail
Bloemfontein	14.29	71.43	28.57	14.29	21.43	71.43
Kimberley	45.45	72.73	9.09	9.09	0	27.27

Ticks were represented by larvae (74.95%) and nymphs (24.66%), while adult stages were only recorded for *Ixodes bakeri*-like ticks. Five tick genera of which 12 species and 8 species groups (including unknown species) were recovered on *M. namaquensis* (Table 2). The most abundant and prevalent ticks belonged to 2 species groups *Rhipicephalus simus*/*follis* (0.70 ± 0.23, 11.11%) and *Haemaphysalis spinulosa* group (0.58 ± 0.20, 15.74%) (Table 3). While the most abundant tick species was *Rhipicephalus neumanni* (0.24 ± 0.14) and *Haemaphysalis elliptica* was the most prevalent (5.56%), *Rhipicephalus follis* was the most widespread (occurred at 4 localities), while the remaining tick species were recorded at fewer localities (1–3 localities) (Tables 4 and 5). A single individual of *Nuttalliella* cf. *namaqua* was recorded at 1 locality (Loeriesfontein).

## Discussion

The study recorded a large diversity of ectoparasites that include 57 ectoparasite taxa (49 species and 8 species groups). This includes several new locality and host records for ectoparasite taxa and reveals the existence of undescribed species. This finding supports the prediction that the current ectoparasite profile of *M. namaquensis* is underestimated. The large diversity is possibly attributed to the opportunistic behaviour of *M. namaquensis* (Macdonald *et al*., 1999; Soliman *et al*., 2001). A similar pattern was recorded for *Rhabdomys pumilio* (4-striped mouse), another opportunistic rodent species in South Africa (Matthee *et al*., 2007, 2010; Froeschke *et al*., 2013; Barnard *et al*., 2015; van der Mescht and Matthee, 2017). In particular, >30 ectoparasite taxa (representing fleas, lice, mesostigmatid and trombiculid mites and ticks) are associated with *R. pumilio* in the southern and western parts of South Africa (Matthee *et al*., 2007, 2010; Froeschke *et al*., 2013; Barnard *et al*., 2015; van der Mescht and Matthee, 2017). Fagir *et al*. (2014) recorded at least 22 ectoparasite taxa on *M. namaquensis* at a single locality in the Savanna biome in South Africa. The latter study was based on 313 *M. namaquensis* individuals trapped seasonally over 12 months.

In the present study, fleas were present on almost 50% of the rodents. *Xenopsylla brasiliensis* and *C. godfreyi* were the most prevalent and abundant flea species, supporting the findings of Fagir *et al*. (2014). Both flea species were characterized by male-biased infestation at most (*C. godfreyi*), or all localities (*X. brasiliensis*). The sex-ratio pattern recorded for *X. brasiliensis* is supported by de Meillon *et al*. (1961) and Fagir *et al*. (2014). However, the pattern recorded for *C. godfreyi* is not supported by previous records (de Meillon *et al*., 1961; Fagir *et al*., 2014) and warrants further research. According to previous records (Segerman, 1995; Fagir *et al*., 2014; van der Mescht and Matthee, 2017), *C. godfreyi*, *E. aganippes* and *Listropsylla aricinae* should have a close association with *M. namaquensis*. However, the low occurrence of *L. aricinae* may be due to an incomplete overlap between the current sampling localities and the flea's preferred geographic distribution (spanning the drier western part of South Africa from the Cape to Namibia) (Segerman, 1995). Four generalist (i.e. broader host preferences) flea species (*D. ellobius*, *Listropsylla agrippinae*, *Listropsylla dorippae* and *X. brasiliensis*) were abundant on *M. namaquensis* in the present study. These fleas are known vectors of plague (causative agent *Yersinia pestis*). *Dinopsyllus ellobius* has been identified as one of the most significant plague vectors in South Africa (Ingram, 1927; de Meillon *et al*., 1961) and both *Dinopsyllus lypusus* (also recorded in the study) and *L. dorippae* are important and efficient vectors for plague in Africa (Heisch *et al*., 1953; Kilonzo and Mhina, 1982, 1983; Makundi *et al*., 2003). *Ctenocephalides felis* is a reservoir and vector of *Rickettsia felis*, which can be transmitted to both humans and animals, including domestic cats (Harasen and Randall, 1986; Greene and Breitschwerdt, 2006; Tsai *et al*., 2009) and this flea can transmit *Bartonella* spp. (Bouhsira *et al*., 2013). Transmission of *Bartonella* is mainly through the feces of infected fleas (Foil *et al*., 1998; Finkelstein *et al*., 2002; Gutiérrez *et al*., 2015).

The 2 louse species recorded on *M. namaquensis*, *H.* cf. *patersoni* and *P. praomydis*, are both known from *M. namaquensis* and the closely related *Aethomys chrysophilus* (red rock rat) (Durden and Musser, 1994; Braack *et al*., 1996; Fagir *et al*., 2014). These host associations are based on morphological characters, although a recent molecular study provides strong evidence that *H.* cf. *patersoni* on *M. namaquensis* is genetically distinct from the same morphotype on *A. chrysophilus* (Bothma *et al*., 2020). It is quite possible that the same holds true for *P. praomydis*, but this remains to be tested (Bothma *et al*., 2020). Cryptic species have been detected in several ectoparasite taxa (Poulin and Keeney, 2008; Malenke *et al*., 2009), including the louse *Polyplax arvicanthis* on the widely distributed *Rhabdomys* genus in South Africa (du Toit *et al*., 2013). The presence of cryptic species among parasites may be a common occurrence as their small body size and morphological stages can cause some difficulty in distinguishing between species based on morphology (Perkins *et al*., 2011). Overall, lice were the most prevalent taxon and showed the highest mean abundance of all the ectoparasite taxa in the present study. Both louse species showed greater prevalence and mean abundance on *M. namaquensis* in the present study (43.06%, 2.94 ± 0.46 and 47.69%, 7.48 ± 1.11, respectively) compared to Fagir *et al*. (2014) (16.2%, 0.89 ± 0.27 and 2.1%, 0.10 ± 0.04). These differences may be due to variation (in study design and parasite removal methods) between the 2 studies. As mentioned, the study by Fagir *et al*. (2014) was conducted seasonally at 1 locality and in addition rodents were visually inspected. In contrast, whole-body examinations were conducted under a stereomicroscope in the present study. Female-biased sex ratios were not a general pattern for either of the 2 louse species. It is possible that louse sex ratios vary seasonally and in association with individual louse infestations on the host. The latter has been recorded for anoplurid lice on humans (Rozsa, 1997) and chewing lice on birds (Rozsa *et al*., 1996; Pap *et al*., 2013). Based on the findings in the present study, the geographic range of the 2 louse species only partially overlaps: *H.* cf. *patersoni* occurred in the central and eastern summer rainfall regions of the country, while *P. praomydis* was more widely distributed across South Africa, which suggests that the latter species is more tolerant of diverse climatic conditions (present in summer and winter rainfall regions).

Mites (in the order Mesostigmata) were recorded on half of all rodents. In all, 3 species, namely *A. rhabdomysi*, *L*. *fritzumpti* and *Laelaps* aff. *grenieri* were the most common mite species. The high prevalence of *A. rhabdomysi* (25.46%) is supported by Fagir *et al*. (2014), who recorded a 20.4% prevalence for *A. rhabdomysi* on *M. namaquensis*. Only 2 mite species (*A. rhabdomysi* and *Androlaelaps zuluensis*) were shared with Fagir *et al*. (2014). In general, the overall dominance of *A. rhabdomysi*, *L*. *fritzumpti* and *L.* aff. *grenieri*, in the present study, was also recorded at the individual localities where they occurred. *Androlaelaps rhabdomysi* occurred at most of the sampling localities. However, based on the per-locality-infestation levels, it does appear that the species prefers the central localities such as Kimberley, Kuruman, Steynsburg and Bethulie. Previous studies on *R. pumilio* also recorded this species in the western and southern regions of South Africa, which suggests that the mite species has a country-wide distribution (Matthee and Ueckermann, 2008; S. Matthee, unpublished data). In the present study, *L. fritzumpti* was absent from the southern and south-western winter rainfall localities and more common in the summer rainfall central and north-eastern localities. There is also no record of this species on 2 other rodent species [*Otomys irroratus* (Southern African vlei rat) and *R. pumilio*] at various localities in the Western Cape Province (Matthee *et al*., 2007, 2010). Host records for *L. fritzumpti* include a range of host species such as *Aethomys* spp., *Elephantulus rupestris* (western rock sengi), *Gerbillurus paeba* (hairy-footed gerbil) and *R. pumilio* (Herrin and Tipton, 1976). The third most prevalent mite, *L.* aff. *grenieri*, was present at 5 localities, of which 4 were located centrally and 1 in the north-eastern region of South Africa. *Laelaps grenieri* (Taufflieb and Mouchet, 1956) has been recorded from a multitude of small mammal hosts, primarily *Lemniscomys* spp. in north-western Africa [Democratic Republic of the Congo (DRC); Zumpt, 1961]. The great geographical distance between the present study and the DRC, as well as morphological inconsistencies between the 2 species, suggests that our mite is different from, but shares resemblances with *L. grenieri* from the DRC. Female-biased infestations were common for the most abundant mite species (*A. rhabdomysi*, *L. fritzumpti* and *L.* aff. *grenieri*) and are in agreement with previous studies on South African rodents [*M. namaquensis* (Fagir *et al*., 2014) and *R. pumilio* (Matthee *et al*., 2007)]. The pattern of female bias is a widespread phenomenon among parasitic mites and may be attributed to the parthenogenetic reproductive systems of mites (Norton *et al*., 1993; Sonenshine, 1993; Matthee *et al*., 2007). In addition, female laelapid mites require more frequent blood meals (for oogenesis) and also use hosts to disperse. This is in contrast to males that feed less and remain in the nest (Radovsky, 1985, 1994). The remaining 8 mesostigmatid mite species appeared to be less common on *M. namaquensis* and have more localized distributions (present at 1–3 localities). Interestingly, 3 species (*A. zuluensis*, *Laelaps muricola* and *Laelaps vansomereni*) were previously recorded on *M. namaquensis* (Zumpt, 1961; Engelbrecht *et al*., 2014). The low occurrence of at least 2 of the remaining species may be due to a preference for other rodent species and/or geographic areas. For example, *Androlaelaps dasymys* was present on *M. namaquensis* at 1 locality (Hammanskraal), but this mite has been recorded on *R. pumilio* and *O. irroratus* in the southern (Stellenbosch, Somerset West, Malmesbury, Grabouw and Swellendam), western (Vanrynsdorp) and north-western (Springbok and Groblershoop) parts of South Africa (Matthee *et al*., 2007, 2010; S. Matthee, unpublished data). It is evident from this study that current information with regard to host and geographic range of mesostigmatid mites on rodents is lacking for South Africa.

Chiggers were present on almost half (41.20%) of all rodents. Fourteen species were recorded of which 2 are new undescribed species and 2 are potentially new species. The discovery of new chigger species on rodents in South Africa is mainly due to past limited research interest in the taxonomic group and the lack of local taxonomic expertise (Barnard *et al*., 2015; Stekolnikov and Matthee, 2019). *Gahrliepia nana*, *Microtrombicula mastomyia* and *S. morosi* have previously been recorded on rodents [e.g. *Aethomys ineptus* (Tete veld rat) and *Saccostomus campestris* (South African pouched mouse)] in the north-eastern Savanna biome in South Africa (Zumpt, 1961; Matthee *et al*., 2020). *Herpetacarus gerrhosauri* and *Hyracarus longipilosus* are known species that are recorded here for the first time after their description on 2 lizard species *Gerrhosaurus flavigularis* (yellow-throated plated lizard) and *Pseudocordylus subviridis* (Drakensberg crag lizard) from Mullers Pass and Witzieshoek Naturelle Reserve, Free State, South Africa for the former and *Procavia capensis* (rock hyrax) from Cedara, KwaZulu-Natal, South Africa for the latter by Lawrence (1949). *Hypotrombidium meleagride* presents the first record outside the type locality, which is Malmesbury in the Western Cape, South Africa (Vercammen-Grandjean and Langston, 1976). *Microtrombicula squirreli* and *Schoutedenichia paraxeri* are recorded in South Africa for the first time as these species were previously recorded from the DRC (Zumpt, 1961; Stekolnikov, 2018). The present study provides the first record of the genus *Kayella* in South Africa. The genus was previously recorded in localities spanning Europe, Asia and North America (Nielsen *et al*., 2021). Lastly, *Tateracarus foliosetosus* and *Tateracarus kimberleyensis* are the second and third species of the previously monotypic genus *Tateracarus*, with the type species *Tateracarus quadrisetosus*, recorded on *Gerbilliscus leucogaster* (bushveld gerbil) in Namibia (Stekolnikov, 2018; Stekolnikov and Matthee, 2022). In the present study, most chigger species were recorded at a single locality. However, 2 species (*H.* aff. *namibiensis* and *S. morosi*) were recorded at multiple localities. Chiggers are regarded as habitat specialists (Shatrov and Kudryashova, 2006) though some species have wider tolerance ranges and can occur in multiple habitat types (Mohr, 1947; Traub and Wisseman, 1974; Matthee *et al*., 2020). Based on the chigger data from 2 localities in the Savanna biome, the ears were the preferred parasitope on *M. namaquensis*. This parasitope has previously been recorded for chiggers, as a group, on *M. namaquensis* in the south-eastern Savanna biome, South Africa (Fagir *et al*., 2014; D. Fagir, personal communication). *Microtrombicula mastomyia* displayed a similar preference for rodent ears in a recent study conducted in the north-eastern Savanna biome in South Africa (Matthee *et al*., 2020). The thickness of the host skin may be an important factor as the cheliceral blades that are used for attachment are minute (e.g. 24 and 35 *μ*m for *Schoutedenichia horaki* and *Ascoschoengastia ueckermanni*, respectively, from South Africa) (Matthee *et al*., 2020). A preference for thin-skinned areas has also been recorded for chiggers on lizards (Arnold, 1982; Goldberg and Holshuh, 1992; Klukowski, 2004). In addition, the ear parasitope provides protection against removal by oral grooming (Goff, 1979; Barnard *et al*., 2015). Similar to previous studies, the tail base was also one of the preferred parasitopes in the present study (Barnard *et al*., 2015; Matthee *et al*., 2020).

Ticks were present on half of all rodents and were represented by 20 taxa of which 12 were identified to species and 8 to species groups (including unknown species). In general, immature life stages (larvae and nymphs) were recorded on *M. namaquensis* (Petney *et al*., 2004; Durden, 2006; Matthee *et al*., 2007, 2010; Horak *et al*., 2018). The morphological characters of larvae and nymphs are such that they can easily be identified to genus level, but these characters are often not sufficiently diagnostic to make a specific diagnosis (Walker *et al*., 2000). As a result, several of the larvae and nymphs were assigned to species groups. The genera, *Rhipicephalus* and *Haemaphysalis* represented most of the tick taxa (species and species groups). The higher species richness recorded for *Rhipicephalus* is supported by Fagir *et al*. (2014), where 6 of the 8 tick taxa were conspecifics within the *Rhipicephalus* genus. It is not possible to make inferences on species groups as they consist of multiple species that may vary in host preference and distribution. In the present study, *R. neumanni* was the most abundant species, but only present at 1 locality (60% prevalence at Kuruman). The Kuruman region is xeric (<340 mm per annum), which is in agreement with the species' observed preference for xeric regions in South Africa (Horak *et al*., 2018). *Micaelamys namaquensis* is a new host record for *R. neumanni* that was previously reported on *A. chrysophilus* (1 larva) and *Mastomys* sp. (1 nymph) in Namibia (Horak *et al*., 2018). In the present study, *Rhipicephalus lunulatus* was recorded at a single locality (Marken) and on <40% of the rodents. This tick species has been recorded previously on *Elephantulus myurus* (eastern rock sengi), *A. chrysophilus* and *M. namaquensis* in South Africa, but in low abundance (1–3 individuals per host species) (Horak *et al*., 2018). *Rhipicephalus follis* was recorded at 4 localities of which 3 fall within the Grassland biome and the fourth (Hammanskraal) in the Savanna. The latter locality presents a new host and biome record for *R. follis* (Horak *et al*., 2018). The low prevalence and abundance of *Rhipicephalus distinctus* in the present study (only recorded at Bethulie) is puzzling as this tick seems to have a preference for hosts that prefer rocky habitats such as *P. capensis*, *E. myurus*, *Elephantulus edwardii* (Cape rock elephant-shrew) and *M. namaquensis* (Horak *et al*., 2018). Furthermore, *R. distinctus* was the most prevalent (67.1%) and abundant tick on *M. namaquensis* in the study by Fagir *et al*. (2014). Several of the localities sampled during winter (June and July) are close to previous sampling records for the species (Horak *et al*., 2018). It is possible that season played a role, as the larval stage appears to be more common during spring and summer (Horak *et al*., 2018). *Rhipicephalus exophthalmos* was recorded at 2 localities (Postmasburg and Steynsburg), with 30% of the rodents at Postmasburg infested. Postmasburg is a new locality record for *R. exophthalmos*, which occurs throughout Namibia and has a patchy distribution in the south-western and southern parts of South Africa (Horak *et al*., 2018). In addition, *M. namaquensis* is a new host recorded as the immature stages seem to have a preference for *Macroscelides proboscideus* (round-eared elephant-shrew), *E. edwardii* and *Lepus saxatilis* (scrub hare) (Horak *et al*., 1991; Fourie *et al*., 2005). *Rhipicephalus warburtoni* was abundant at 3 localities (Alldays 1, Hammanskraal and Steynsburg) that fall within the known distribution range of the species in South Africa. This tick is commonly associated with rocky outcrops and hosts that frequent these habitats. *Rhipicephalus warburtoni* has previously been recorded on *M. namaquensis* (Fagir *et al*., 2014), although it seems to prefer hares and *E. myurus* as hosts (Fourie *et al*., 2005; Harrison *et al*., 2012; Fagir *et al*., 2015). *Hyalomma truncatum* has a country-wide distribution and displays low host-specificity, with adults being generalists while immature stages are present on a range of small and medium-sized mammals such as *Lepus capensis* (Cape hares), *L. saxatilis* and murid rodents (Horak *et al*., 1991, 1993, 1995, 2018; Matthee *et al*., 2007). Studies on *R. pumilio* have recorded *H. truncatum* mainly from drier areas (e.g. Oudtshoorn) (S. Matthee, unpublished data). A similar pattern was recorded in the present study, with the species only recorded from localities with a mean annual rainfall <349 mm (Kuruman, Kimberley and Loeriesfontein). *Haemaphysalis elliptica* has been recorded throughout South Africa's Savanna, Grassland, Thicket, Fynbos and Succulent Karoo biomes (Horak *et al*., 2018). Adult life stages prefer carnivores while the immature stages seem to prefer rodent hosts, particularly *R. pumilio* and *M. namquensis* but are also present on other small mammals (Fourie *et al*., 1992; Petney *et al*., 2004; Matthee *et al*., 2007). *Nuttalliella* cf. *namaqua* was represented by a single individual (larva) recorded from a single rodent at Loeriesfontein in the Northern Cape. It is possible that this tick is *N. namaqua*, a monotypic species within the family Nuttalliellidae. This is based on the fact that *M. namaquensis* is regarded as a preferred host for *N. namaqua* and the latter species has been recorded in regions close and with similar climatic conditions to Loeriesfontein (Mans *et al*., 2011; Horak *et al*., 2012; Apanaskevich, 2021). In addition to the immature stages, 2 adult female *I. bakeri*-like ticks were collected. Although species in the genus *Ixodes* usually displays a strong on-host female bias (Horak *et al*., 2018), very few adult ticks were collected to confirm this in the present study. Several of the tick species that were recorded in the study are known vectors for disease-causing pathogens or are directly responsible for causing disease. In particular, *H. elliptica* is a vector of *Babesia rossi*, the causative agent for canine babesiosis in South Africa (Matjila *et al*., 2004, 2008). *Hyalomma truncatum* is known to transmit *Babesia caballi* to horses (de Waal, 1990), and females of certain strains of *H. truncatum* may secrete toxins in their saliva, which may cause sweating sickness in cattle, with greater risk for calves (Neitz, 1956). *Hyalomma truncatum* is also a vector of Crimean-Congo haemorrhagic fever virus to humans (Hoogstraal, 1979; Swanepoel *et al*., 1987). In Zimbabwe, *R. lunulatus* has been associated with tick paralysis in calves and lambs. The adults of *R. neumanni* occur between the claws of sheep and may cause lameness and the formation of abscesses (Walker, 1990). Lastly, *R. warburtoni* has been reported as causing paralysis in new-born Angora goats (Horak *et al*., 2018).

The current study in addition to published literature highlights the rich diversity of ectoparasite taxa that are associated with the opportunistic and regionally widespread *M. namaquensis* (Table 7). Several of the tick and flea species are of medical and/or veterinary importance and it is therefore important to monitor the population sizes of *M. namaquensis* in anthropogenic habitats. Further, the study makes novel contributions in terms of new locality and host records for several ectoparasite and undescribed chigger species. Data on the chigger diversity and mite and louse geographic distribution provide valuable baseline information for future studies on rodent ectoparasites.

**Table 7.** Summary of ectoparasite taxa associated with *Micaelamys namaquensis* (previously *Aethomys namaquensis*) to date

Parasite taxon	Parasite species	Reference
Fleas		
	*Chiastopsylla carus*	Edwards *et al*. (1974)
	*Chiastopsylla coraxis*	van der Mescht and Matthee (2017)
	*Chiastopsylla gariepensis*	Segerman (1995)
	*Chiastopsylla godfreyi*	Fagir *et al*. (2014); van der Mescht and Matthee (2017); current study
	*Chiastopsylla mulleri simplex*	van der Mescht and Matthee (2017)
	*Chiastopsylla nama*	van der Mescht and Matthee (2017)
	*Chiastopsylla octavii*	van der Mescht and Matthee (2017); current study
	*Chiastopsylla quadrisetis*	van der Mescht and Matthee (2017)
	*Chiastopsylla rossi*^a^	van der Mescht and Matthee (2017)
	*Praopsylla powelli*	van der Mescht and Matthee (2017)
	*Ctenopsyllus aganippes*	Segerman (1995)
	*Ctenocephalides calceatus*^a^	van der Mescht and Matthee (2017)
	*Ctenocephalides felis*^a^	Segerman (1995); current study
	*Dinopsyllus ellobius*^a^	Fagir *et al*. (2014); van der Mescht and Matthee (2017); current study
	*Demeillonia granti*	Fagir *et al*. (2014)
	*Dinopsyllus lypusus*	Segerman (1995); current study
	*Epirimia aganippes*	Fagir *et al*. (2014); van der Mescht and Matthee (2017); current study
	*Hysophthalmus temporis*	van der Mescht and Matthee (2017)
	*Listropsylla agrippinae*	van der Mescht and Matthee (2017); current study
	*Listropsylla aricinae*	van der Mescht and Matthee (2017); current study
	*Listropsylla dorippae*^a^	Segerman (1995); current study
	*Xenopsylla brasiliensis*^a^	Fagir *et al*. (2014); van der Mescht and Matthee (2017); current study
	*Xenopsylla cheopis*^a^	van der Mescht and Matthee (2017)
	*Xenopsylla eridos*^a^	van der Mescht and Matthee (2017)
	*Xenopsylla versuta*	Segerman (1995)
Lice		
	*Hoplopleura aethomydis*	Durden and Musser (1994); Fagir *et al*. (2014)
	*Hoplopleura* cf. *patersoni*	Current study
	*Hoplopleura patersoni*	Fagir *et al*. (2014)
	*Polyplax praomydis*	Fagir *et al*. (2014); current study
Mesostigmatid mites		
	*Androlaelaps dasymys*	Till (1963); current study
	*Androlaelaps rhabdomysi*	Fagir *et al*. (2014); current study
	*Androlaelaps marshalli*	Till (1963); Fagir *et al*. (2014)
	*Androlaelaps zuluensis*	Till (1963); Fagir *et al*. (2014); current study
	*Androlaelaps zumpti*	Till (1963)
	*Laelaps brandbergensis*	Taufflieb (1954)
	*Laelaps fritzumpti*	Taufflieb (1964); current study
	*Laelaps* aff. *grenieri*	Current study
	*Laelaps liberiensis*	Taufflieb (1964); current study
	*Laelaps muricola*	Zumpt (1961); current study
	*Laelaps roubaudi*	Fagir *et al*. (2014)
	*Laelaps simillimus*	Fagir *et al*. (2014)
	*Laelaps vansomereni*	Zumpt (1950); current study
	Macronyssidae sp.	Shepherd and Narro (1983)
	*Ornithonyssus roseinnesi*	Shepherd and Narro (1983); current study
	*Myocoptes* sp.	Current study
Chiggers		
	*Afropolonia tgifi*	Goff (1983)
	*Acomatacarus thallomyia*	Stekolnikov (2018)
	*Ascoschoengastia* spp.	Malan (2015)
	*Austracarus* spp.	Malan (2015)
	*Gahrliepia nana*	Stekolnikov (2018); current study
	*Herpetacarus aethomys*	Stekolnikov (2018)
	*Herpetacarus gerrhosauri*	Current study
	*Herpetacarus longispinus*	Stekolnikov (2018)
	*Hypotrombidium meleagride*	Current study
	*Hyracarus* aff. *Namibiensis*	Current study
	*Hyracarus lawrencei*	Stekolnikov (2018)
	*Hyracarus longipilosus*	Current study
	*Hyracarus* sp.	Current study
	*Kayella* sp.	Current study
	*Leptotrombidium muridium*	Malan (2015)
	*Microtrombicula mastomyia*	Current study
	*Microtrombicula squirreli*	Current study
	*Neoschoengastia* spp.	Malan (2015)
	*Odontacarus* spp.	Malan (2015)
	*Schoengastia* spp.	Malan (2015)
	*Schoutedenichia morose*	Current study
	*Schoutedenichia paraxeri*	Current study
	*Schoutedenichia* sp.	Current study
	*Tateracarus foliosetosus*	Stekolnikov and Matthee (2022)
	*Tateracarus kimberleyensis*	Stekolnikov and Matthee (2022)
	*Trombicula* spp.	Malan (2015)
	*Zumptrombicula misonnei*	Goff (1983); Stekolnikov (2018)
Ticks		
	*Haemaphysalis aciculifer-*like	Current study
	*Haemaphysalis elliptica* group	Harrison *et al.* (2011); current study
	*Haemaphysalis elliptica*^a^	Harrison *et al.* (2011); current study
	*Haemaphysalis elliptica/colesbergensis*	Harrison *et al.* (2011); current study
	*Haemaphysalis leachi* group	Fourie *et al*. (1992); Horak *et al*. (2005); current study
	*Haemaphysalis spinulosa* group	Fourie *et al*. (1992); Fagir *et al*. (2014); current study
	*Hyalomma marginalum rufipes*	Fourie *et al*. (1992)
	*Hyalomma truncatum*^a^	Horak *et al*. (2005); current study
	*Ixodes bakeri-*like	Current study
	*Ixodes rubicundus*^a^	Fourie *et al*. (1992); Horak *et al*. (2005)
	*Nuttalliella namaqua*	Horak *et al*. (2012)
	*Nuttalliella* cf. *namaqua*	Current study
	*Rhipicephalus appendiculatus*^a^	Fagir *et al*. (2014)
	*Rhipicephalus decoloratus*^a^	Fagir *et al*. (2014)
	*Rhipicephalus distinctus*	Fourie *et al*. (1992); Fagir *et al*. (2014); current study
	*Rhipicephalus evertsi evertsi*	Fourie *et al*. (1992); Horak *et al*. (2005); Fagir *et al*. (2014)
	*Rhipicephalus exophthalmos*	Keirans *et al*. (1993); current study
	*Rhipicephalus follis*	Fourie *et al*. (1992); Horak *et al*. (2005); current study
	*Rhipicephalus follis*/*gertrudae*	Fourie *et al*. (1992); Horak *et al*. (2005); current study
	*Rhipicephalus gertrudae*	Fourie *et al*. (1992); Horak *et al*. (2005); current study
	*Rhipicephalus lunulatus*	Harrison *et al*. (2011); current study
	*Rhipicephalus neumanni*	Current study
	*Rhipicephalus pravus* group^a^	Keirans *et al*. (1993); Walker *et al*. (2000); current study
	*Rhipicephalus punctatus*	Fourie *et al*. (1992)
	*Rhipicephalus simus*/*distinctus*	Horak *et al*. (2005); current study
	*Rhipicephalus simus*/*follis*	Horak *et al*. (2005); Harrison *et al*. (2011); current study
	*Rhipicephalus warburtoni*/*arnoldi*	Horak *et al*. (2005); Fagir *et al*. (2014)
	*Rhipicephalus warburtoni*^a^	Horak *et al*. (2005); Fagir *et al*. (2014); current study

a Species of veterinary and medical importance.

## Data Availability

All data generated or analysed during this study are included in this published article. The datasets used and/or analysed are available from the corresponding author upon reasonable request.

## Supplementary material

For supplementary material accompanying this paper visit https://doi.org/10.1017/S0031182022000750. click here to view supplementary material
