# Supplementary material for: Diversity and distribution of ectoparasite taxa associated with Micaelamys namaquensis (Rodentia: Muridae), an opportunistic commensal rodent species in South Africa
Source: Parasitology. 2022 Jun 1;149(9):1229–48. doi: 10.1017/S0031182022000750 (PMC10090637; doi:10.1017/S0031182022000750)
Supplement: Supplementary file 1 [file S0031182022000750sup001.docx]

# Stevens et al

# Supplementary material

Table S1. Sex ratio of the four most prevalent flea species on *Micaelamys namaquensis* (n = 216) at multiple localities in South Africa during 2017-2018.

| Localities | | | | | | | | | | | |
| --- | --- | --- | --- | --- | --- | --- | --- | --- | --- | --- | --- |
| Species | Across localities | Alldays 1 | Alldays 2 | Bethulie | Bloemfontein | Hammanskraal | Kimberley | Marken | Postmasburg | Steynsburg |  |
| *Chiastopsylla godfreyi* | 1:0.72 | - | - | 1:0.82 | 1:0.38 | - | 1:0.5 | - | - | 1:1 |  |
| *Dinopsyllus ellobius* | 1:0.64 | 2:0 | - | - | 1:1.33 | 1:0.25 | 1:1 | - | - | - |  |
| *Epirimia aganippes* | 1:1.39 | 1:1.25 | - | 1:2 | - | - | - | - | - | 1:1.33 |  |
| *Xenopsylla brasiliensis* | 1:0.84 | 0:2 | 1:2 | - | 1:0.5 | 1:0.77 | 1:0.8 | 1:1 | 1:0.89 | - |  |

Table S2. Sex ratio of the two louse species on *Micaelamys namaquensis* (n = 216) at multiple localities in South Africa during 2017-2018.

| Localities | | | | | | | | | | | | | |
| --- | --- | --- | --- | --- | --- | --- | --- | --- | --- | --- | --- | --- | --- |
| Species | Across  Localities | Alldays 1 | Alldays 2 | Bethulie | Bloemfontein | De Doorns | Hammanskraal | Kimberley | Kuruman | Loeriesfontein | Marken | Postmasburg | Steynsburg |
| *Hoplopleura* cf. *patersoni* | 1:1.40 | 1:1.2 | 1:1.71 | 1:0.96 | 1:1.32 | - | 1:2.06 | 0:1 | 1:0.33 | - | 1:2.39 | 1:0.68 | 1:1.80 |
| *Polyplax praomydis* | 1:0.75 | - | 1:3.33 | 1:0.76 | 1:4.5 | 1:0.5 | - | 1:1.41 | 1:1 | 1:2.75 | - | 1:0.59 | 1:0.67 |

Table S3. Sex ratio of the three most prevalent mite species on *Micaelamys namaquensis* (n = 216) at multiple localities in South Africa during 2017 - 2018.

|  |  | Localities | | | | | | | | |  |  |  |
| --- | --- | --- | --- | --- | --- | --- | --- | --- | --- | --- | --- | --- | --- |
| Species | Across  Localities | Alldays 1 | Alldays 2 | Bethulie | Bloemfontein | De Doorns | Hammanskraal | Kimberley | Kuruman | Loeriesfontein | Marken | Postmasburg | Steynsburg |
| *Androlaelaps rhabdomysi* | 1:2.30 | - | 1:1 | 1:3.81 | 1:3 | 0:2 | - | 1:1.33 | 1:0.4 | - | 1:0 | 1:2 | 1:1.56 |
| *Laelaps fritzumpti* | 1:6.05 | - | 1:18 | 1:8 | 3:0 | - | 1:0 | 1:4.38 | - | - | 0:3 | 0:10 | 1.63 |
| *Laelaps* aff. *grenieri* | 1:3.91 | - | - | 1:1.25 | 1:1.67 | - | 1:9.67 | 1:1 | - | - | - | - | 0:3 |
